# Abnormal Elevation of Anti-Mullerian Hormone and Androgen Levels Presenting as Granulosa Cell Tumor

**DOI:** 10.3389/fonc.2021.641166

**Published:** 2021-03-22

**Authors:** Hongbin Chi, Ning Huang, Huamao Liang, Rong Li, Congrong Liu, Jie Qiao

**Affiliations:** ^1^ Center for Reproductive Medicine, Department of Obstetrics and Gynecology, Peking University Third Hospital, Beijing, China; ^2^ Department of Obstetrics and Gynecology, Peking University Third Hospital, Beijing, China; ^3^ Department of Pathology, Peking University Third Hospital, Beijing, China

**Keywords:** anti-Mullerian hormone, granulosa cell tumor, hyperandrogenism, infertility, ovarian cyst

## Abstract

We report a rare subtype of adult cystic granulosa cell tumor (AGCT) characterized by elevated anti-Mullerian hormone and hyperandrogenism. A 35-year-old woman with primary infertility, hyperandrogenism, and irregular menses who was previously diagnosed with polycystic ovarian syndrome was diagnosed with AGCT based on histopathological examination and FOXL2 genetic test after laparoscopy. Due to fertility aspirations, she underwent controlled ovarian stimulation followed by embryo cryopreservation before salpingo-oophorectomy, and two embryos were frozen-thawed and transferred after surgery. A healthy female infant was delivered at 40 weeks’ gestation. Cystic granulosa cell tumors should be considered a differential diagnosis in patients with persistent ovarian cysts and hyperandrogenism. Younger patients with AGCT with fertility goals should consider active assisted reproduction measures to preserve fertility before treatment for AGCT.

## Introduction

Adult granulosa cell tumor (AGCT) of the ovary is a rare type of ovarian tumor that originates from ovarian sex-cord stromal cells and represents approximately 3–5% of malignant ovarian tumors (1). Patients with AGCTs often present with irregular menses and symptoms of virilization such as acne, hirsutism, and alopecia. AGCT may present as cystic, solid, or solid-cystic tumors on ultrasonography or pathological examination (2, 3). Patients with cystic granulosa cell tumors characterized by excessive androgen secretion tend to have a poor prognosis (3). Both serum inhibin and anti-Mullerian hormone (AMH) are elevated in patients with AGCT and can be used as tumor markers for diagnosing AGCT; however, AMH is a more valuable diagnostic tool as its elevation is constant throughout the menstrual cycle (4).

Serum AMH levels are a marker of ovarian reserve that are commonly measured in patients seeking infertility treatment. AGCT is rarely considered in the differential diagnosis of patients with elevated AMH levels; instead, patients with elevated AMH levels and hyperandrogenism are more likely to be diagnosed with polycystic ovarian syndrome (PCOS), which is much more common. Since the treatment of reproductive cancers is likely to destroy a patient’s ovarian reserve and trigger premature ovarian insufficiency, early diagnosis of AGCT is crucial in infertile patients of childbearing age so that active fertility preservation can be performed. Fertility preservation is an effective treatment that offers hope of conception and pregnancy for patients with cancer, which improves their quality of life and alleviates feelings of regret and depression after cancer treatment (5). Unfortunately, studies regarding the safety and efficacy of fertility preservation in patients with AGCT are lacking and only a few studies states the safety and outcomes in patients with ovarian cancer (6, 7).

We report the management of a patient with AGCT characterized by infertility, abnormally elevated AMH levels, and hyperandrogenism. This report provides a therapeutic strategy for AGCT treatment and fertility preservation for patients with primary infertility.

## Case

**Figure 1:**
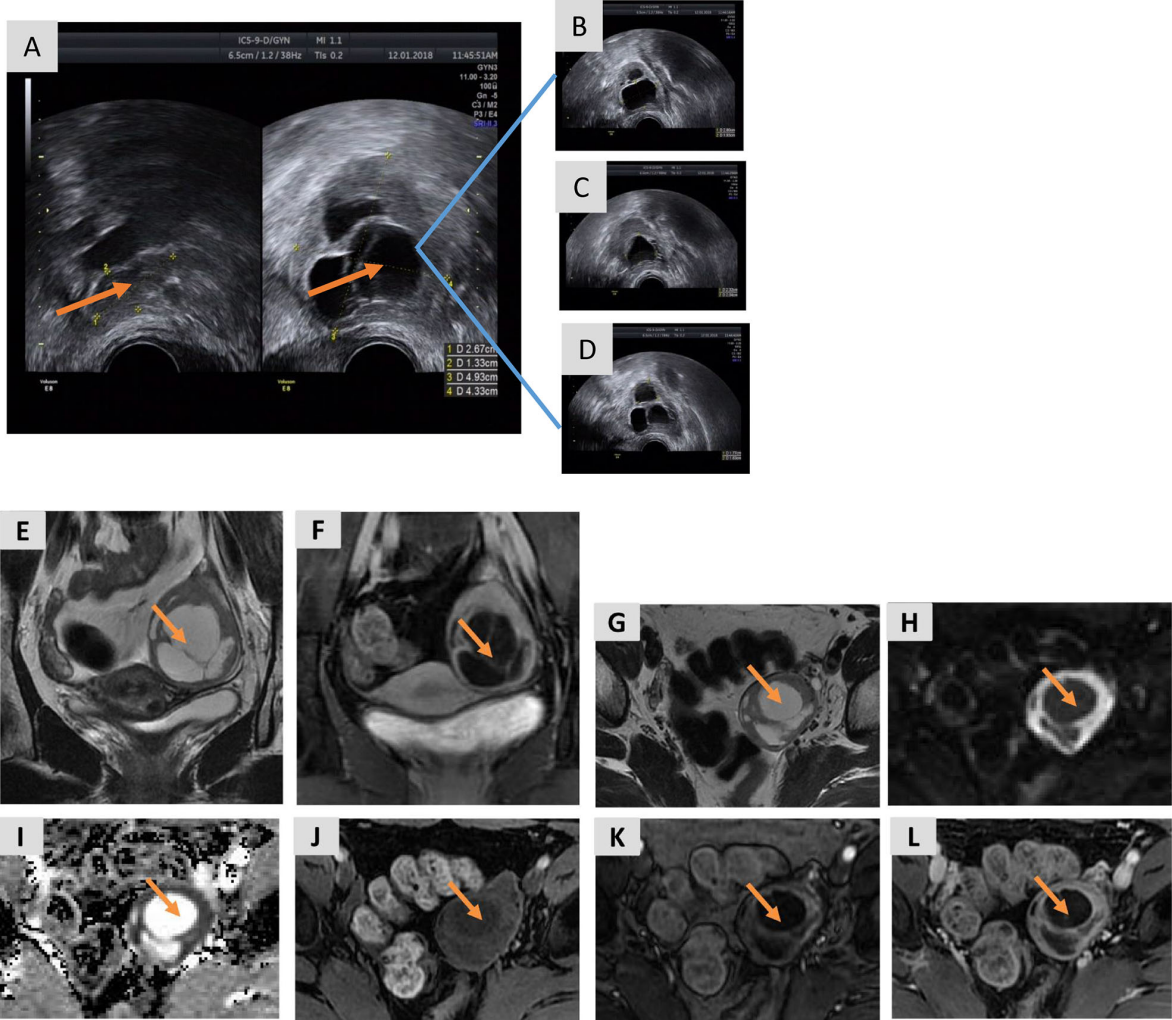
Transvaginal ultrasound images and MRI of the pelvis prior to ovarian cystectomy. **(A)** The right ovary is normal, measuring 2.7 × 1.3 cm with 2–3 antral follicles, while the left ovary is enlarged, measuring 4.9 × 4.3 cm with 2–3 antral follicles. **(B–D)** The three cysts in the left ovary measure 2.8 × 1.9 cm **(B)**, 2.3 × 2.0 cm **(C)**, and 1.8 × 1.6 cm **(D)**. **(E)** A coronal T2-weighted image shows a large, multicystic mass in the left ovary with a slightly hyperintense, solid component of the septa and a thickened wall. **(F)** A coronal T1-weighted image with fat saturation of the delayed phase obtained after gadolinium administration shows marked enhancement of the solid components of the tumor. **(G)** An axial T2-weighted image shows a large, multicystic mass of the left ovary with septa and a thickened wall. **(H**, **I)** DWI (b=1,000) (Panel **H**) and the ADC map **(I)** show restricted diffusion of the solid components. **(J)** An axial T1-weighted image with fat saturation before enhancement shows hypo-intensity of the lesion. **(K)** An axial T1-weighted image of the arterial phase obtained after gadolinium administration shows marked early enhancement of the solid component of the tumor. **(L)** An axial T1-weighted image with fat saturation of the delayed phase obtained after gadolinium administration shows persistent enhancement of the solid component of the tumor.

A 35-year-old female with a 6-year history of primary infertility presented to our clinic for fertility treatment. The patient had a history of oligomenorrhea without dysmenorrhea, and normal secondary sexual characteristics. She had a normal body mass index (19.07 kg/m^2^), and she had hirsutism with no acne. Her partner was 37 years old and healthy with normal sperm concentration and motility. The couple engaged in a normal frequency of sexual intercourse and had no history of sexual dysfunction. Bimanual palpation failed to reveal any abnormalities of the uterus and right adnexa; however, the left adnexa was mildly enlarged. Transvaginal ultrasonography demonstrated an anteverted uterus of normal size and 2–3 antral follicles in the right ovary. The left ovary was enlarged, measuring 4.9 × 4.3 cm with 2–3 antral follicles. There were three separate, irregularly shaped cysts located in the left ovary measuring 2.8 × 1.9 cm, 2.3 × 2.0 cm, and 1.8 × 1.6 cm (**Figure 1**). The patient was diagnosed in another hospital with PCOS 3 years prior due to hyperandrogenism and irregular menses and undergone four failed cycles of ovulation induction therapy with an aromatase inhibitor (letrozole), including one failed cycle of intrauterine insemination. Growth of a dominant follicle was observed by ultrasonography during treatment, and 2–3 cysts measuring 1–3 cm in diameter were persistently visible in the left ovary. The nonspecific tumor markers CA125, CA19-9, AFP, and CEA were within the normal range.

Serum AMH levels were measured to further evaluate ovarian reserve. AMH was significantly elevated (52.8 ng/mL; normal range: 0.24–11.78 ng/mL in women aged 20–40 years old). AMH, gonadotropin, and steroid sex hormone levels were reassessed on the second day of the next menstrual cycle (**Table 1**), revealing a persistently elevated AMH level (24.1 ng/mL). Subsequently, the patient developed amenorrhea, and her AMH on day 47 of amenorrhea was 35.2 ng/mL. The patient also had significantly elevated luteinizing hormone (LH) and testosterone (T). Magnetic resonance imaging (MRI) and ultrasonography failed to show any abnormalities in the pituitary gland, adrenal glands, or thyroid.

**Table 1:** Hormone profile from presentation to post-salpingo-oophorectomy.

Date	LMP	PRL^a^	FSH^b^	LH^c^	E2^d^	T^e^	AND^f^	PRG^g^	AMH^h^
		(ng/ml)	IU/L	IU/L	pmol/L	nmol/L	nmol/L	nmol/L	ng/ml
2018-1-19	2018-1-4								52.8
2018-2-6	2018-2-5	9.63	1.29	8.96	119	0.87	2.26	0.64	
2018-2-9	2018-2-5								24.1
2018-3-22	2018-2-5	7.77	5.63	33.4	178	5.41	6.93	1.6	35.2
2018-3-29	2018-2-5	7.43	5.72	39.5	113	6.03	7.42	1.2	
2018-5-30	2018-2-5	5.09	4.56	30.3	141	6.17	7.98	1.47	
**2018-7-10: Left Ovarian Cystectomy**									
2018-7-20	2018-2-5	24.5	4.42	46.8	2786	1.12	10.2	5.69	2.68
2018-7-27	2018-2-5	21.4	1.26	2.85	2217	1.24	11.4	80.5	
2018-8-6	2018-8-5	16.8	8.91	4.85	246	1.01	8.86	1.45	1.63
2018-9-7	2018-9-2	8.74	7.69	9.15	151	<0.69	4.06	1.1	
2018-9-27	2018-9-26								0.69
2018-10-26	2018-9-26								0.49
2018-11-17	2018-9-26								<0.06
**2018-9-11: Left Salpingo-oophorectomy**									
2019-2-23	2019-2-22	15.5	2.63	2.2	521	<0.69	3.75	0.71	<0.06
2019-4-17	2019-4-16	9.19	12	3.16	227	<0.69	3	0.79	
2019-5-14	2019-5-13	15.3	5.45	3.42	111	<0.69	2.9	0.75	
2019-6-17	2019-6-8								0.11
2019-8-29	2019-7-5								0.30

a Reference range (ng/ml): 1.9-25.

b Reference range (IU/L): 2.8-11.3, follicular phase; 5.8-21, ovulatory phase; 1.2-9.0, luteal phase.

c Reference range (IU/L): 1.1-11.6, follicular phase; 17-77, ovulatory phase; 0-14.7, luteal phase.

d Reference range (pmol/L): 0-587, follicular phase; 124-1468, ovulatory phase; 110-905, luteal phase.

e Reference range (nmol/L): 0-2.53.

f Reference range(nmol/L): 1.0-11.5.

g Reference range (nmol/L): ND-3.6, follicular phase; 1.5-5.5, ovulatory phase; 3.0-68, luteal phase.

h Reference range: 0.24–11.78 ng/mL in women aged 20–40 years old.

LMP, last menstrual period; PRL, prolactin; FSH, follicle-stimulating hormone; LH, luteinizing hormone; E2, estradiol; T, testosterone; AND, androstenedione; PRG, progesterone.

**Figure 2:**
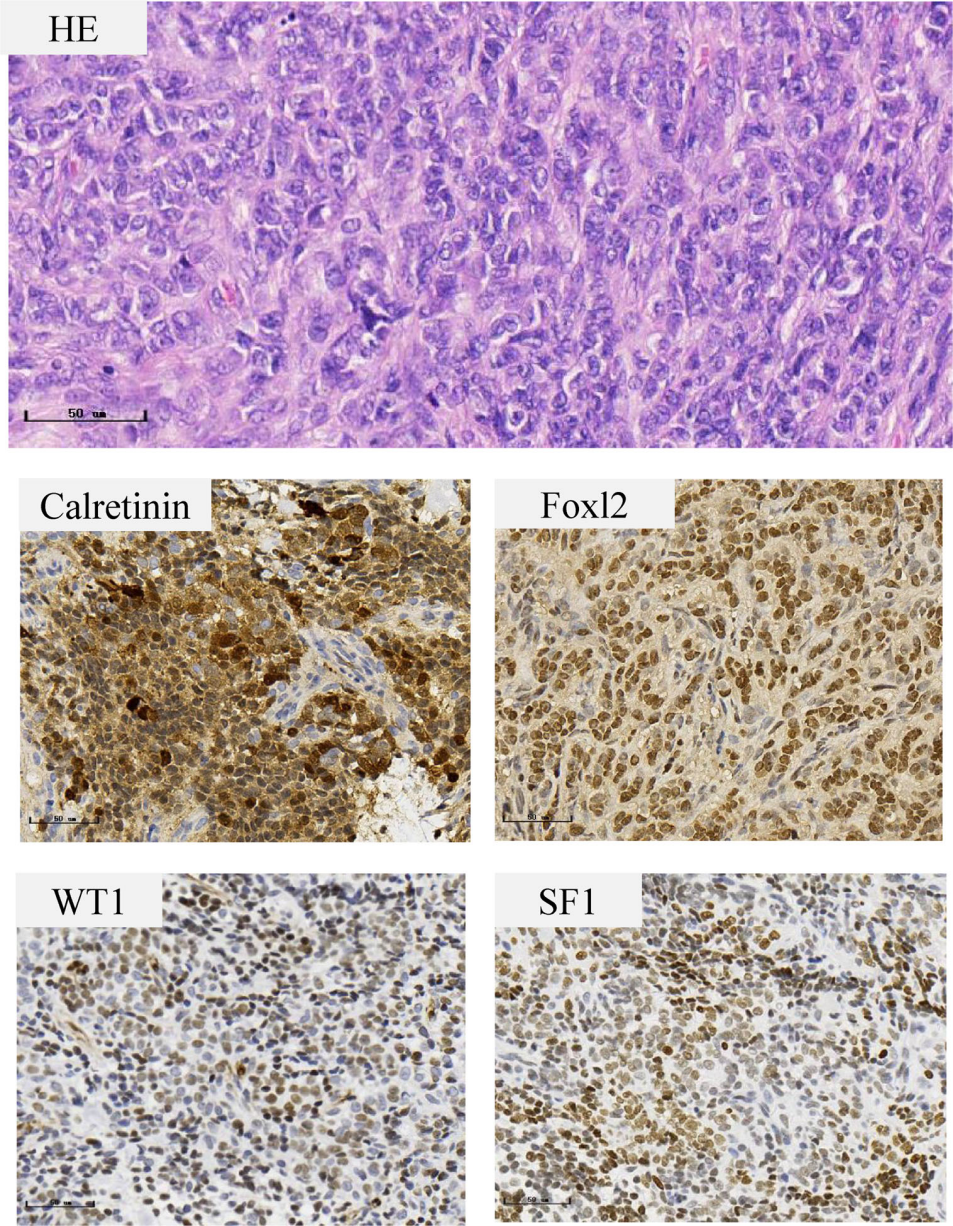
Histopathological features of the ovarian granulosa cell tumor. The tumor cells are small with round to oval nuclei with a fine chromatin pattern, inconspicuous nucleoli, and scanty cytoplasm. Immunohistochemical staining show positive staining in Calretinin, Foxl2, WT1 and SF1.

A preoperative MRI of the pelvis revealed a mass in the left adnexa measuring 4.6 × 3.8 × 4.9 cm with significant enhancement of the cyst wall and septum (**Figure 1**). These findings suggested a left ovarian tumor. Laparoscopy revealed no significant abnormalities in the uterus, right ovary, bilateral fallopian tubes, other viscera, or peritoneum. The left ovary measured approximately 4 × 3 × 2 cm and contained some thin-walled cystic lesions which ruptured during the left ovarian cystectomy. Three connected cysts with diameters of 1–2 cm contained a crystal serous fluid. Yellow granular tissue was attached to the inner wall of the cyst, which was brittle and similar to a corpus luteum. The inner wall of the cyst was removed in its entirety and sent for a frozen section. The postoperative paraffin-embedded section confirmed the diagnosis of AGCT (**Figure 2**). Immunohistochemical examination showed positive FOXL2, SF-1, CD99, and WT-1, with focal positivity for calretinin, inhibin-α, and CK mixture. Approximately 5% of cells in the hotspot region were positive for Ki-67, and the specimen was negative for CK7 and EMA. The specimen was found to harbor the characteristic FOXL2 c.402C>G (p.C134W) mutation *via* Sanger sequencing. This mutation is found in approximately 90% of AGCTs (**Figure S1**). AMH and gonadal hormone concentrations were measured after surgery (**Table 1**).

Due to the early stage of the tumor, the patient’s age, and her desire for fertility preservation, a unilateral salpingo-oophorectomy with comprehensive surgical staging was considered to minimize the risk of recurrence in the residual left ovary. However, the patient had poor ovarian reserve with insufficient antral follicle counts and decreased AMH (1.63 ng/mL), and this was expected to further decline after the salpingo-oophorectomy and chemotherapy. Therefore, the patient underwent controlled ovarian stimulation followed by embryo cryopreservation prior to the salpingo-oophorectomy.

Gonadal hormone levels were measured, and a transvaginal ultrasound was performed on day 2 of the menstrual cycle before initiating the process of ovarian stimulation. The hormone levels are shown in **Table 1**. The ultrasound indicated 1–2 follicles on each ovary. Ovarian stimulation was performed using an aromatase inhibitor (letrozole) combined with a gonadotropin and gonadotropin-releasing hormone (GnRH) antagonist. Letrozole was initiated at a dosage of 5 mg/day on the second day of the menstrual cycle for 3 days before gonadotropin administration and then continued with the gonadotropin. GnRH antagonist was administered when the size of the leading follicle reached 14 mm to prevent premature ovulation. Human chorionic gonadotropin (HCG) combined with 0.2 mg of the GnRH agonist was administered when two follicles reached 20 mm in diameter. Five oocytes were retrieved 36 h after HCG administration and were fertilized by intracytoplasmic sperm injection. Three embryos were successfully cultivated and cryopreserved.

A laparoscopic unilateral salpingo-oophorectomy with peritoneal and omental multipoint biopsies was performed after fertility preservation. The postoperative pathology reported that no tumor tissue was detected in the left ovary, biopsy tissues, or abdominal rinses. The patient received four courses of chemotherapy (TC regimen: paclitaxel combined with carboplatin), and a GnRH agonist was administered during chemotherapy.

Two months after the patient’s last chemotherapy treatment, the patient underwent frozen-embryo transfer (FET). The patient’s AMH level was < 1 ng/mL, and the hormone levels had returned to normal 5 months after the last chemotherapy (**Table 1**). A natural cycle FET was selected to avoid the systemic impact of excessive hormones triggered by an artificial cycle. Two embryos were transferred, and dydrogesterone combined with progynova was used for luteal support. The patient’s HCG level was 1,905 mIU/mL 15 days after FET, and she experienced a small amount of vaginal bleeding 19 days after FET when the HCG level was 8,414 mIU/mL. Transvaginal ultrasonography showed an anechoic mass measuring 0.9 × 0.5 cm located inside the uterine cavity. The patient was administered 2,000 IU HCG. However, the patient experienced mild but sustained vaginal bleeding accompanied by mild abdominal pain 31 days after FET. Transvaginal ultrasonography indicated a gestational sac measuring 4.7 × 1.1 cm inside the uterine cavity with a visible fetal heartbeat and a mass measuring 1.4 × 1.2 cm in the right adnexa (**Figure S2**). Thirty-six days after FET, the patient’s HCG levels increased to 179,175 mIU/mL, with persistent vaginal bleeding. The intrauterine gestational sac and right adnexal mass increased to 3.5 × 2.6 cm and 3.6 × 1.6 cm, respectively (**Figure S2**). A heterotopic pregnancy was considered, although the possibility of tumor recurrence could not be ruled out. Laparoscopy was performed. No abnormalities were identified on the surface of the liver, stomach, intestine, or omentum. The uterus was enlarged to approximately 8 weeks gestation. The left adnexa was absent without evidence of tumor, and the right ovary was normal in appearance. A 2 cm bluish-purple, enlarged mass was found in the isthmus portion of the right fallopian tube. Right salpingectomy was performed, and the postoperative pathology revealed a fallopian tube pregnancy. The HCG level was 326,566 mIU/mL on the second postoperative day. Transvaginal ultrasonography showed a gestational sac measuring 5.2 × 3.7 cm implanted in the uterine cavity a week after the surgery. Subsequently, the patient underwent routine obstetrical care, and a healthy female infant was delivered at 40 weeks’ gestation.

This study was approved by the Peking University Third Hospital Medical Science Research Ethics Committee and a written informed consent has been obtained from the patient.

## Discussion

We report a patient with AGCT with primary infertility, hyperandrogenism, and irregular menses, who was previously diagnosed with PCOS. AGCT originates from granulosa cells, a class of hormone-secreting cells capable of producing estradiol, progesterone, inhibin, and AMH. Thus, patients with AGCT often present with menstrual disturbances (8). Excessive estrogen secretion is the most common finding in patients with AGCT. In this patient, the estrogen level prior to surgery was in the normal follicular-phase range, while LH and T were significantly elevated, a similar profile to that observed in patients with PCOS. However, transvaginal ultrasonography failed to identify morphological features consistent with polycystic ovary. The AMH level in this patient was 8–10-fold higher than that of age-matched healthy women, which was inconsistent with the number of antral follicles.

Elevated AMH and LH levels as well as hyperandrogenism are frequently present in infertile women with PCOS, a common endocrine disease often diagnosed by reproductive specialists. AMH, a dimeric glycoprotein specifically produced by granulosa cells of small growing follicles, plays an important role in regulating folliculogenesis (9). Many studies have demonstrated that preantral and small antral follicles secrete the majority of AMH found in the circulation and AMH has been shown to be proportional to the number of premature developing follicles in the ovaries; thus, AMH is used to assess ovarian reserve (10, 11). Serum AMH concentrations are 2–4-fold higher in patients with PCOS than in healthy age-matched women and correlate significantly with the increased number of small antral follicles (12). Previous studies have reported the role of a high AMH concentration in the diagnosis of AGCT. Michael et al. monitored serum AMH levels during a 54-month follow-up of a patient diagnosed with a sex-cord tumor and demonstrated that the serum concentration of AMH correlated with the degree of AGCT growth and tumor recurrence (13). Rey et al. evaluated the AMH level in patients with AGCT and other types of ovarian cancers and confirmed that AMH is a specific and sensitive serum marker for the diagnosis of AGCT and monitoring both AMH and inhibin B during follow-up of a patient with AGCT improves the detection of recurrent disease (14).The absence of inhibin measurement is one of the limitations of our study, combined testing with AMH and inhibin B in such patients should be recommended.

The source of elevated LH and T remains unknown. Previous studies have suggested that a deficiency of aromatase may suppress the conversion of androgen to estrogen, thus triggering excessive androgen accumulation (3). Recent studies have identified a specific subset of hypothalamic GnRH-positive neurons that express AMH receptors both in mice and humans. These studies showed that excess AMH triggers an imbalance in the hypothalamic–pituitary–ovarian axis, subsequently inducing hyperandrogenism and elevated LH levels *via* binding to the AMH receptors of GnRH neurons (15, 16). The results of those studies suggest that exposure to high levels of AMH may generate high LH and T levels in a GnRH-dependent manner. However, the elevation of LH and T is indeed perplexing; therefore, the definite mechanism needs more studies. After undergoing a salpingo-oophorectomy, the patient’s LH and T levels normalized and the AMH decreased. These changes in hormone levels indicate that AMH is crucial for diagnosing ovarian hormone-secreted tumors and provide clinical support for the regulation of the GnRH-LH signaling pathway by AMH.

In addition to the disturbance in hormone regulation observed in this patient, 2–3 cysts with an average diameter of 1–3 cm were persistently detected on ultrasonography which did not significantly increase in size over a 3-year period. The differentials include growing follicles, functional ovarian cysts, ovarian endometriotic cysts, or ovarian tumors. Pelvic MRI is an effective tool for distinguishing cysts based on their unique characteristics. In our patient, an MRI revealed a left adnexal mass with significant enhancement of the cyst wall and septum on the enhanced scan, suggesting an ovarian tumor. Based on the clinical, laboratory, and imaging data, we firstly suspected a stromal tumor. A panel of immunohistochemical markers combined with FOXL2 genetic tests after ovarian cystectomy confirmed the diagnosis of AGCT (17).

Surgery is the standard therapy for AGCT, and a unilateral salpingo-oophorectomy combined with postoperative chemotherapy was considered for this patient who was diagnosed with stage Ic AGCT (18). However, the patient was young with poor ovarian reserve after the ovarian cystectomy and wished to preserve her fertility. Since further decline in the ovarian reserve was inevitable after the planned salpingo-oophorectomy and chemotherapy, it was unlikely that she would be able to conceive spontaneously or through assisted reproductive technology after the second surgery. After a comprehensive assessment of the patient’s circumstances, fertility preservation was performed before the salpingo-oophorectomy and chemotherapy.

In this ovarian stimulation protocol, letrozole was administered based on a micro-stimulation protocol. Letrozole is an aromatase inhibitor with the ability to suppress endogenous estrogen production and was recommended for fertility preservation in women with hormone-sensitive cancers (19). Considering the risk of blastocyst culture, all three frozen embryos were day 3 embryos, and their quality was rated as grade 1. Two embryos were successfully thawed and transferred to increase the pregnancy rate, as the pregnancy rate of day 3 embryos is lower than that of blastocysts (20, 21). However, a heterotopic pregnancy occurred. The incidence of heterotopic pregnancy has gradually increased with the widespread use of assisted reproductive technology (22). The risk factors for heterotopic pregnancy are similar to those associated with ectopic pregnancy. When comparing heterotopic versus intrauterine twin pregnancy after embryo transfer, studies have shown that previous ectopic pregnancy, tubal surgery, pelvic surgery, and pelvic infection are significant risk factors for the occurrence of heterotopic pregnancy (23, 24). Several studies have revealed a lower risk of ectopic pregnancy in frozen-thawed blastocyst transfer than in day 3 transfer and fresh transfer. Further, the risk of ectopic pregnancy is lower in pregnancies following single embryo transfers than double embryo transfers (25–28). This patient underwent two operations before FET, placing her at an increased risk of an ectopic or heterotopic pregnancy. Thus, a single frozen-thawed blastocyst transfer may be a better choice for patients with a history of pelvic surgery.

In summary, AGCT is an uncommon ovarian malignancy that lacks distinctive clinical symptoms and pathological signs. Patients with AGCT present with a mass on pelvic examination, which may be confused with ovarian endometrioma on transvaginal ultrasound. Furthermore, AGCT can be diagnosed as PCOS due to abnormal AMH and sex hormone levels. Therefore, including AGCT on a differential diagnosis is essential. In this case report, AGCT was diagnosed based on a significantly elevated AMH level, confirming that this elevation of AMH is a reliable serum marker for diagnosing AGCT. Surgery is the mainstay of AGCT treatment and is necessary for obtaining tissue for pathological diagnosis and performing precise tumor staging. However, the ovarian reserve may be destroyed after surgery. Fertility preservation and a unilateral salpingo-oophorectomy can be considered in women of childbearing age with careful tumor staging and the exclusion of extra-ovarian cancer. A micro-stimulation protocol and a single embryo transfer may provide the best fertility results; however, more research is needed.

## Data Availability Statement

The original contributions presented in the study are included in the article/**Supplementary Material**. Further inquiries can be directed to the corresponding author.

## Ethics Statement

The studies involving human participants were reviewed and approved by Peking University Third Hospital Medical Science Research Ethics Committee. The patients/participants provided their written informed consent to participate in this study. Written informed consent was obtained from the individual(s) for the publication of any potentially identifiable images or data included in this article.

## Author Contributions

HC and NH wrote the initial draft of the paper, and all authors contributed to the manuscript revision. HL performed operation and provided imaging of operation and MRI. CL made the histopathologic diagnosis. HC, NH, RL and JQ took part in the diagnosis and treatment with fertility preservation of AGCT in this patient. All authors contributed to the article and approved the submitted version.

## Funding

This work was supported by National Key R&D Program of China (Grant No. 2019YFC1005106) and National Natural Science Foundation of China (Grant no. 81871212).

## Conflict of Interest

The authors declare that the research was conducted in the absence of any commercial or financial relationships that could be construed as a potential conflict of interest.
