# Supplementary material for: Abnormal Elevation of Anti-Mullerian Hormone and Androgen Levels Presenting as Granulosa Cell Tumor
Source: Front Oncol. 2021 Mar 22;11:641166. doi: 10.3389/fonc.2021.641166 (PMC8019939; doi:10.3389/fonc.2021.641166)
Supplement: Supplementary file 1 [file DataSheet_1.docx]

Supplementary Material

# Supplementary Tables

| Date | Days for Gn | LH (IU/L) ^a^ | E2 (pmol/L) ^b^ | PRG (nmol/L) ^c^ |
| --- | --- | --- | --- | --- |
| 2018-8-6 | 1 | 4.85 | 246 | 1.45 |
| 2018-8-12 | 7 | 6.18 | 466 | 1.34 |
| 2018-8-14 | 9 | 2.8 | 892 | 1.98 |
| 2018-8-15 | 10 | 3.01 | 1832 | 2.49 |

**Supplementary Table 1. Data of hormone changes during ovarian stimulation therapy.**

Abbreviation: Gn, gonadotropin; LH, luteinizing hormone; E2, estradiol; PRG, progesterone;

a. Reference range (IU/L): 1.1-11.6, follicular phase; 17-77, ovulatory phase; 0-14.7, luteal phase

b. Reference range (pmol/L): 0-587, follicular phase; 124-1468, ovulatory phase; 110-905, luteal phase

c. Reference range (nmol/L): ND-3.6, follicular phase; 1.5-5.5, ovulatory phase; 3.0-68, luteal phase

# Supplementary Figures


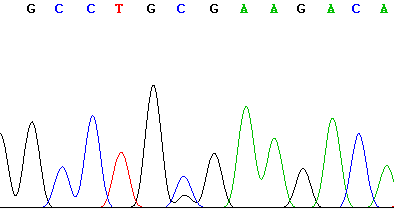


**Supplementary Figure 1. Sanger sequencing of FOXL2 gene**

By Sanger sequencing, this patient was diagnosed with the characteristic FOXL2 c.402C>G (p.C134W) mutation.


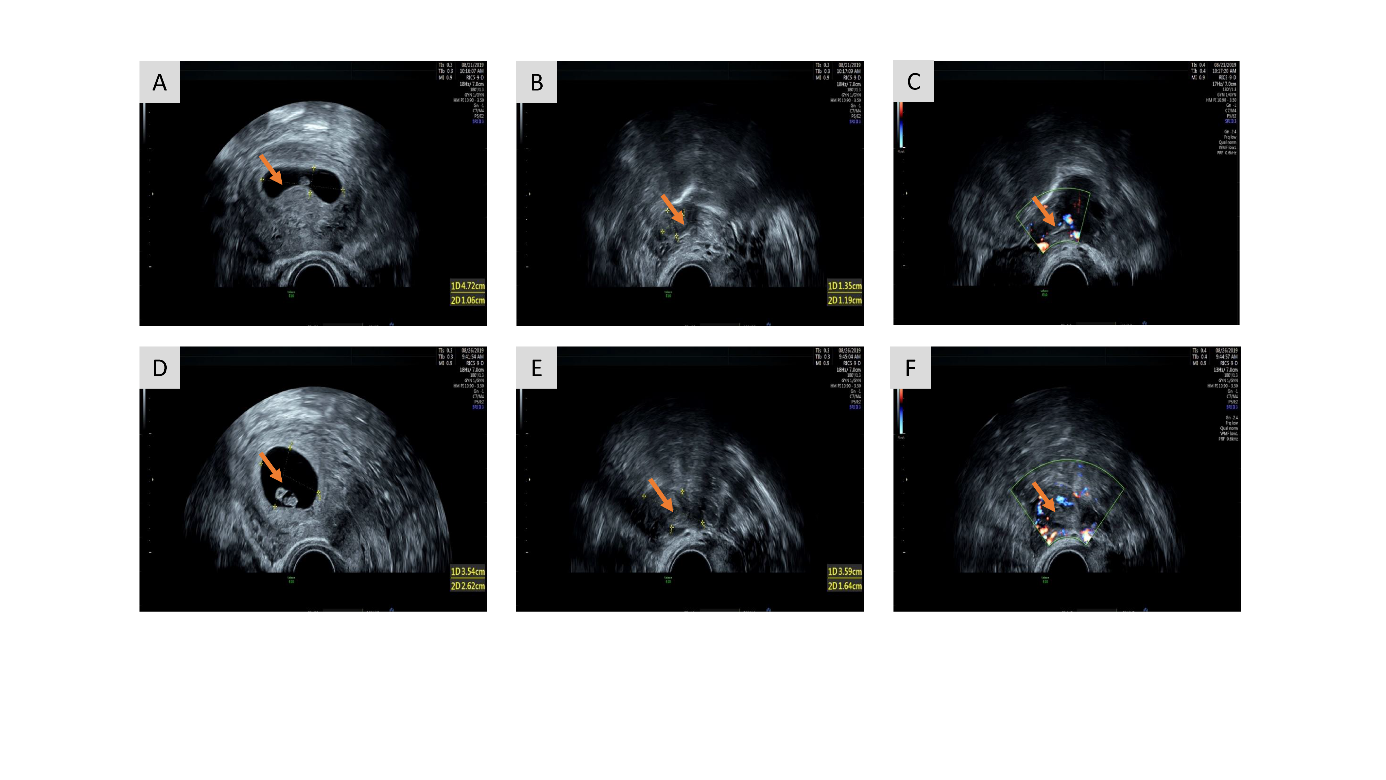


**Supplementary Figure 2. Transvaginal ultrasound image after FET**

Panels A, B, and C were performed 31 days after FET. A: A gestational sac measuring 4.7 × 1.1 cm is detected inside the uterine cavity with a visible fetal heartbeat. B and C: A mass measuring 1.4 × 1.2 cm is shown in the right adnexa with blood flow signaling. Panels D, E, and F were performed 36 days after FET. D: An intrauterine gestational sac measuring 3.5 × 2.6 cm is shown. E and F: An enlarged increased right adnexal mass measuring 3.6 × 1.6 cm with blood flow signaling is shown.

Abbreviation: FET, frozen embryo transfer


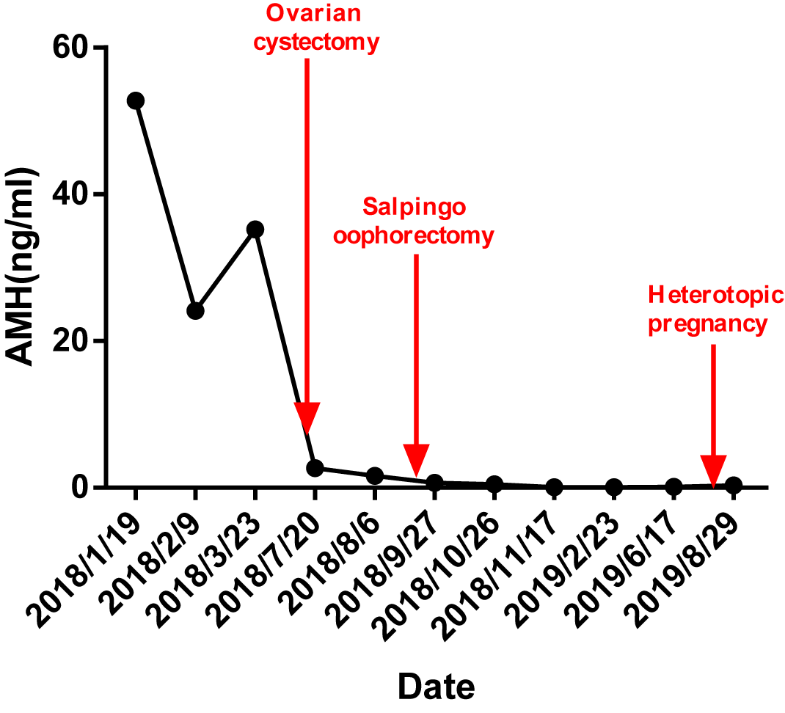


**Supplementary Figure 3. Dates of surgical resection and corresponding changes in AMH levels**

The serum concentration of AMH rapidly decreased and remained stable after ovarian cystectomy.

Abbreviation: AMH, anti-Mullerian hormone


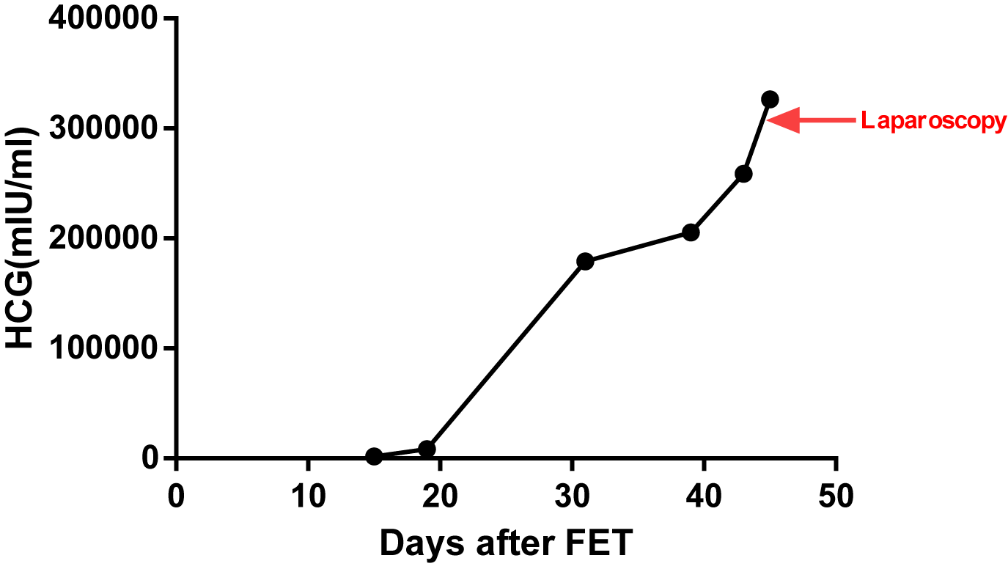


**Supplementary Figure 4. HCG profile after FET**

The arrow denotes the time of right salpingectomy by laparoscopy.

Abbreviation: HCG, human chorionic gonadotropin; FET, frozen embryo transfer
